# Information Processing in Social Insect Networks

**DOI:** 10.1371/journal.pone.0040337

**Published:** 2012-07-16

**Authors:** James S. Waters, Jennifer H. Fewell

**Affiliations:** School of Life Sciences, Arizona State University, Tempe, Arizona, United States of America; Universitat Rovira i Virgili, Spain

## Abstract

Investigating local-scale interactions within a network makes it possible to test hypotheses about the mechanisms of global network connectivity and to ask whether there are general rules underlying network function across systems. Here we use motif analysis to determine whether the interactions within social insect colonies resemble the patterns exhibited by other animal associations or if they exhibit characteristics of biological regulatory systems. Colonies exhibit a predominance of feed-forward interaction motifs, in contrast to the densely interconnected clique patterns that characterize human interaction and animal social networks. The regulatory motif signature supports the hypothesis that social insect colonies are shaped by selection for network patterns that integrate colony functionality at the group rather than individual level, and demonstrates the utility of this approach for analysis of selection effects on complex systems across biological levels of organization.

## Introduction

Capturing the essence of biological networks is among the most important challenges facing modern science. Gene regulation, motor control, developmental specialization, and metabolic allometry all emerge as the result of integrated networks. These networks operate at different biological levels but all distribute and transform localized information into larger scale processes [1]–[4]. However, not all biological networks develop or evolve around higher order function. Social networks, the broad class of networks characterizing human and animal social groups, are typically thought to exhibit global-structure consistent with the predictions of generative network models such as preferential attachment [5], [6]. In these systems, interactions benefit and reinforce an individual’s own role within the network [7], but at a potential cost to higher-level properties such as efficiency or resilience [8].

Although generally clustered into one category, social networks can describe many different types of complex systems from aggregations to cohesive social units. Network analyses show global similarities across social systems; they are generally decentralized and scale-free, with network structure emerging from local interactions in the absence of an external controller. However, the function of interactions within social groups should vary with the evolutionary and ecological contexts in which the group evolves. The social interactions within, for example, a pod of dolphins [9], [10] or extended family groups of ground squirrels [11], should serve very different functions than the communication networks among workers within a eusocial insect colony [12]–[15].

Social insect colonies are the hallmark of integrated social units, exhibiting some of the most awe-inspiring examples of complexity in the biological world. Nest architecture that promotes environmental stability [16], division of labor that scales with colony size [17], and collective decision making [18] all take place in the absence of hierarchical control [19]. Social insect communication systems, which include such diverse modalities as direct individual contact, trophallaxis, and broadcast pheromonal signaling, show they are highly regulated units with coordinated individual behaviors that generate emergent effects which are beneficial to the group as a whole [20]. If network structure reflects biological function, then the structure of a social insect colony should vary distinctly from that of social networks generated from associations based on individual success.

We investigate network motif profiles of seed harvester ant colony interaction networks to determine whether their antennation patterns are predominantly random, regulatory, or social in nature. Since the purpose of antennation by ants is to obtain information, the structure of their communication networks is critical to how colonies function [12]. Motif analysis determines the predominant local interaction patterns (3-node directed subgraph motifs) making up a network [21] and has the potential to identify fundamental interaction signatures within networks of different size or context that may correspond to differences in functionality [22]. Previous work by Milo and his colleagues [21], [22] has shown that biological regulatory networks have predominant interaction patterns that move information directionally, while social networks develop bidirectionally-connected cliques as individuals mutually strengthen associations with their neighbors. We ask whether these different types of subgraph representation allow us to differentiate between networks selected for at the individual-level and networks that emerge as a result of group-level selection [8].

## Methods

### Ant Colonies

Whole colonies of *Pogonomyrmex californicus* were reared in the laboratory [17], [23] in artificial nest enclosures (242 cm^2^) containing separate nest and foraging arenas, water tubes, and foraging material including fruit flies, grass seeds, and finch seeds. All adult workers and queens within each of two colonies were uniquely marked. Color codes were applied to the dorsal surface of the ant head, mesosoma, and gaster with fine-tip oil-based paint markers. Ants did not exhibit adverse reactions to the paint or increased mortality following paint marking.

After having been paint-marked, colonies were given two weeks to acclimate to their new markings and the experimental arena, a well-lit lab bench in an observation room maintained at 30 degrees C. A foam pad beneath the nest enclosures dampened vibrations and a sheet of transparent plastic was placed over the nest enclosures to prevent disturbance induced by experimenter exhalation. Fifteen minutes before video-recording, colonies were gently stimulated to engage in work (division of labor) with the addition of foraging items and debris through small openings in the nest enclosure lids. Following these methods, nearly all individuals within the colonies were visible from above and workers within the colonies were observed engaging in normal behaviors including foraging, brood care, food processing, refuse removal, policing, and allogrooming.

### Video Recording

We recorded digital video of colonies within nest enclosures to carefully observe the behaviors and patterns of interactions among individual ants (Movie S1). Video data were recorded using a CCD camera (Flea 2, Point Grey Research, Richmond, BC, Canada) and a 16 mm fixed focal length lens (Edmund Optics, Barrington, NJ, USA) positioned on a copy stand above colony nest enclosures. Uncompressed AVI video (1624×800 pixels, 15 frames per second) was recorded using FlyCapture SDK (Point Grey Research, Richmond, BC, Canada). The arrangement of these components resulted in a resolution of 73.8 pixels per centimeter, more than sufficient to observe the fine-scale antennation patterns between interacting ants. We recorded each colony for a duration of two hours (approximately 550 GB for each recording).

### Networks

To establish networks of directed contacts from the video recordings, each individual ant was tracked throughout the entire recording and her contacts with other ants manually recorded. Contact occurred if the ant stopped and placed both antennae onto another ant, orienting the head towards the contacted ant. Antennation was chosen as the focal behavior because it is a direct form of information exchange and can be clearly characterized ethologically. Networks of colony interactions were constructed as adjacency lists, each individual ant treated as a node, with their directional interactions supplying the network edges. A total of 12 networks were constructed, 5 for colony pcp07-40 and 7 for colony pcp07-35.

All social network data are snapshots of a system in time. For the data to be meaningful, they should be based on a time interval long enough to capture the behavior of the system at a point in time without being so long that behavioral variation over time averages and dampens away relevant interaction patterns. Data to populate the interaction networks in this study were based on the behaviors observed during 26-second subsets of the video recordings for each colony. Analyzing less time than this would have meant that the networks were highly fragmented (i.e., not connected). Preliminary data suggested that reviewing 13–26 seconds of behavior would be sufficient to capture interactions for greater than 90% of the active individuals within the colonies. Of the 12 networks we analyzed, there was an average of 3.17 connected components per network and the largest connected cluster contained on average 92.96% of the nodes in each network. The effects of analysis and observation time on social network structure were investigated by cumulatively pooling networks. For each of the two colonies, we analyzed the network motif representation of networks based on 26, 52, 78, 104, and 130 seconds by combining observations to build sequentially larger networks.

### Motif Analysis

To test hypotheses about the mechanisms responsible for generating colony-level functionality, we analyzed the local-scale structure of interaction networks using triad motif analysis [21], [22]. The primary question addressed by motif analysis is whether particular subgraphs appear more often in an observed network than would be expected in similarly sized networks generated based on the assumptions of specific null models.

Using the implementation of motif analysis executed by Fast Network Motif Detection (FANMOD) [24] we tested the structure of our networks against a network model that randomized the interactions between individuals. The null-model random graphs were generated with the same degree distribution as observed in the colonies to preserve global network structure. Nodes in the random networks also maintained the same number and directionality of edges as in the respective observed networks. The frequencies of each of the 13 directed three-node subgraphs (Text S1) were calculated both for each observed network (N = 12) and the simulated random graphs (N = 10,000 per observed network).

The statistical significance of each subgraph representation within an observed network was calculated by comparing subgraph densities (the ratio of the number of occurrences of a specific subgraph to the total number of subgraphs within a network) between observed and random networks. We estimated bootstrapped p-values calculated as ratio of the number of randomized networks in which the subgraph density was higher than observed to the total number of randomized networks for each subgraph in each observed network. Significantly over-represented subgraphs (p<0.05 and density >0.01) are referred to as network motifs [21]. It is possible that specific subgraphs are not generated within the randomized networks, resulting in cases for which the p-values are undefined. The only subgraph for which this occurred was ID = 13, a subgraph identified in 5/12 networks, but with a instance count greater than one in only two networks and never with a subgraph density greater than 0.01.

Network visualizations and additional descriptive network statistics were generated in R using the igraph package [25], [26]. Degree distributions for the nodes within a network can be modeled as power laws, *p(k) ∝ k^-alpha^*, where *p(k)* is the fraction of vertices having degree *k* and *alpha* is the scaling exponent. We estimated the exponent associated with in-, out-, and total-degree distributions using the methods of both ordinary least squares on log-transformed data and discrete maximum-likelihood estimation of the power-law distribution [27], [28]. Unless described otherwise, data in the results section are presented as means ± standard errors.

## Results

**Figure 1:**
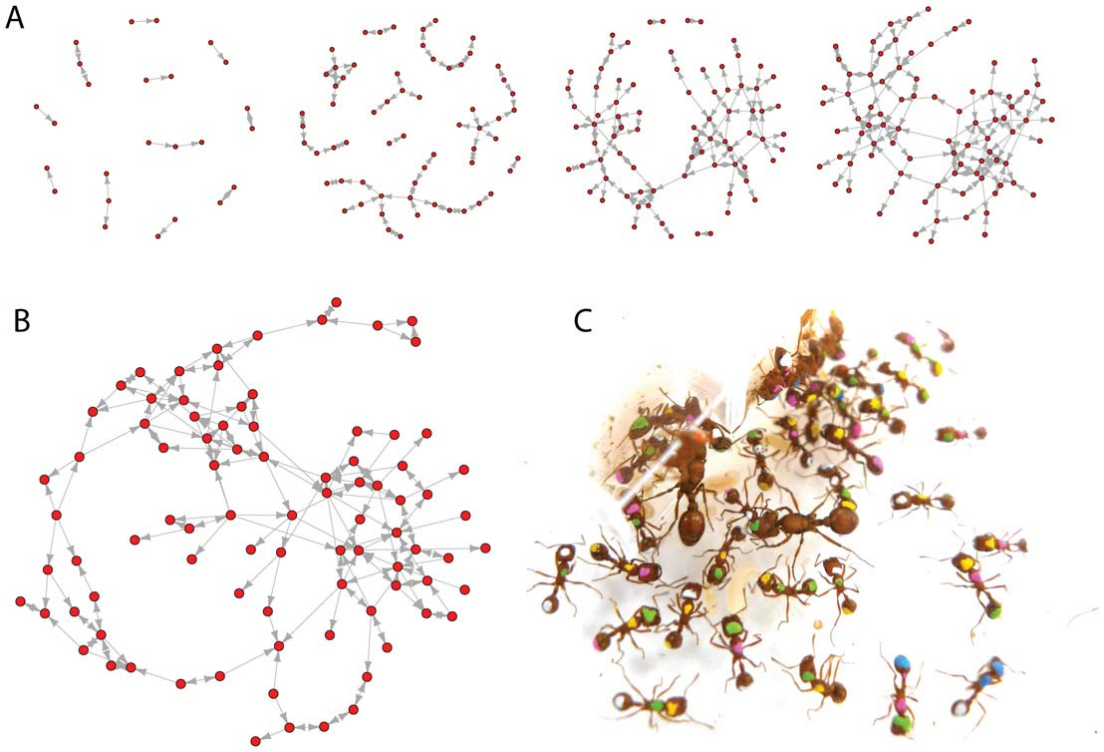
Ant colony interaction networks. (**A**) The development of a directed network of interactions between workers in a single *P. californicus* colony over a period of 60 s. Nodes represent individual workers or queens within a colony and arrows represent interactions between individuals. (**B**) Example *P. californicus* interaction network based on 26 s of colony behavior. (**C**) Photograph of queens and workers within a seed harvester colony; individuals have been painted with unique color combinations to track their interactions.

Seed harvester colony interaction networks (Figure 1) developed at a rate of 4.86±0.08 interactions per ant per minute (Table 1). Networks were composed of an average of 89.17±3.96 nodes and 191.5±18 edges. While differences in colony size affected the number of nodes (F_1,10_ = 19.38) and edges (F_1,10_ = 23.29), there were not significant differences in network topology. Across the 12 networks, the average in-degree power-law exponent was 1.93±0.13 (Table 2) and the average out-degree power-law exponent was 2.03±0.08 (Table 3). There was no significant effect of source colony on in-degree (F_1,10_ = 0.152, p = 0.71) or out-degree (F_1,10_ = 1.77, p = 0.21) exponents and there was also not a significant difference between these exponents (F_1,22_ = 0.387, p = 0.54). The exponents estimated by OLS were less than those estimated by maximum likelihood (in-degree: 3.18±0.08, out-degree: 3.12±0.09), but both sets of estimates are qualitatively consistent with right-skewed degree distributions characteristic of scale-free networks (Text S2).

**Table 1:** Summary statistics for *P. californicus* interaction networks.

Network Statistic	Mean (N = 12)	Standard Deviation
Nodes	89.167	13.730
Edges	191.5	62.372
Average Node Degree	4.213	0.824
Maximum Node Degree	13.333	2.964
Average Path Length	5.256	0.986
Diameter	14.75	2.261
Density	0.024	0.003

This table summarizes global-scale network statistics for the twelve observed *P. californicus* interaction networks.

**Table 2:** Summary of out-degree scaling analysis.

Colony	Slope^1^	SE	R squared	P-value
1	−2.302233	0.2453528	0.9362024	0.0001
2	−1.495037	0.4009239	0.7766024	0.0203
3	−1.993387	0.397453	0.80741	0.0024
4	−2.076154	0.1107807	0.9859641	0
5	−1.718984	0.3488862	0.8585369	0.0079
6	−2.129866	0.2474018	0.9251065	0.0001
7	−1.93115	0.2274315	0.9231746	0.0001
8	−2.371344	0.3693494	0.8918231	0.0014
9	−2.230362	0.3129172	0.8788996	0.0002
10	−2.343456	0.3201249	0.9146595	0.0007
11	−1.773185	0.4323595	0.7061254	0.0046
12	−2.045588	0.2359468	0.8930656	0

(1) This is the OLS-estimated slope for the relationship describing how the number of nodes with a given number of out-degree edges scales with out-degree. The data (x) were transformed prior to regression according to log_10_(x+1). The absolute value of the slope is an estimate for the degree distribution power law exponent (alpha).

**Table 3:** Summary out in-degree scaling analysis.

Colony	Slope^1^	SE	R squared	P-value
1	−1.589522	0.95259	0.258183	0.1337
2	−2.390163	0.4221644	0.8423322	0.0013
3	−1.27468	0.9519083	0.1831009	0.2173
4	−2.434398	0.335663	0.8976089	0.0003
5	−1.105925	1.0091694	0.1305242	0.305
6	−2.685515	0.3487973	0.936789	0.0015
7	−1.742611	0.3006386	0.8936109	0.0044
8	−1.849658	0.3015067	0.8827247	0.0017
9	−1.957485	0.2508555	0.9383577	0.0015
10	−1.838498	0.2517909	0.8555716	0
11	−2.018978	0.4390072	0.7790092	0.0037
12	−2.344854	0.4034605	0.8491615	0.0011

(2) This is the OLS-estimated slope for the relationship describing how the number of nodes with a given number of in-degree edges scales with in-degree. The data (x) were transformed prior to regression according to log_10_(x+1). The absolute value of the slope is an estimate for the degree distribution power law exponent (alpha).

**Figure 2:**
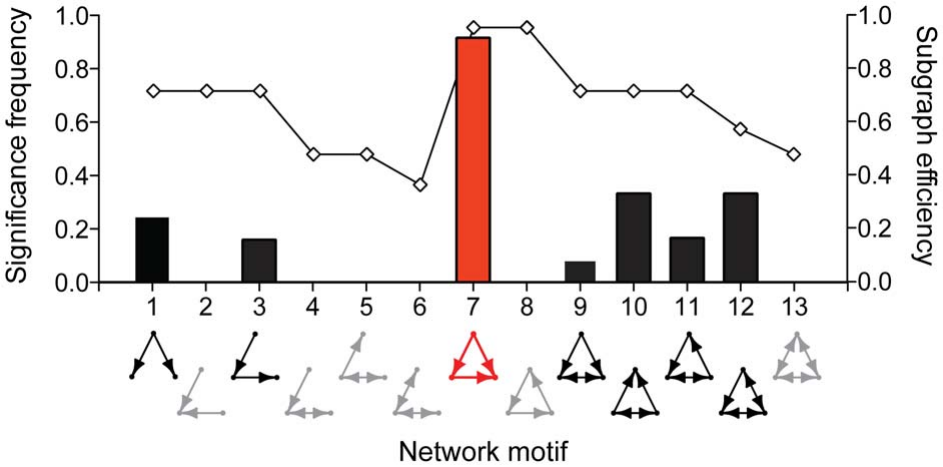
Distribution of network motifs. Network motif analysis deconstructs a network into its constituent subgraphs and determines whether any of these local-scale interaction patterns are represented more frequently than expected for a randomized network of the same size. The subgraphs that were identified as significant motifs in our analysis of social insect colony networks are plotted above in a summary histogram with relative frequencies on the left axis. The interaction efficiencies of each subgraph are plotted as a line with units along the right axis. One of the two subgraphs with the highest efficiencies, the feed-forward loop (motif 7), was also the most dominant motif observed across the *P. californicus* interaction networks. Gray subgraphs were not classified as network motifs, black indicates a subgraph identified as a motif within at least one network, and red indicates the only motif that was identified across the majority of networks.

We used motif analysis to identify the relative significance of the thirteen possible directed subgraphs among every connected triad of ants within our recorded networks (Figure 2). Subgraphs were classified as significant motifs when the frequency of a given subgraph was higher than expected compared to a null model of degree-preserved randomized interaction and its subgraph density was at least 0.01 (Table 4). Eight subgraphs (IDs: 1, 3, 6, 7, 9–12) were classified as motifs in at least one of the 12 observed networks, and one motif, the feed-forward loop (ID: 7), was present in 11/12 networks. The high frequency on significance for the feed-forward loop (i.e. significantly higher expected frequency in each network) indicates it to be a consistent network signature within the colonies we measured.

**Table 4:** Network motif analysis results.

Subgraph ID	Average Observed Density	Observed Networks^1^	Motifs (count >1)^2^	Motifs (density >1%)^3^
1	2.33E-01	12	3	3
2	1.25E-01	12	0	0
3	2.55E-01	12	2	2
4	1.17E-01	12	0	0
5	1.74E-01	12	0	0
6	4.07E-02	12	0	0
7	2.33E-02	11	11	11
8	4.43E-03	9	4	0
9	5.53E-03	11	4	1
10	9.62E-03	11	10	4
11	7.71E-03	11	7	2
12	8.21E-03	12	7	4
13	4.76E-03	5	2	0

This table summarizes the classification of subgraphs as network motifs. (1) The number of observed networks containing each respective subgraph. (2) The number of networks in which the observed density for a subgraph is significantly greater than its density in the random networks and in which the subgraph appears more than once in the observed network. (3) The number of networks in which the average observed density for a subgraph is significantly greater than its density in the random networks and in which the subgraph density is at least one percent in the observed network.

**Figure 3:**
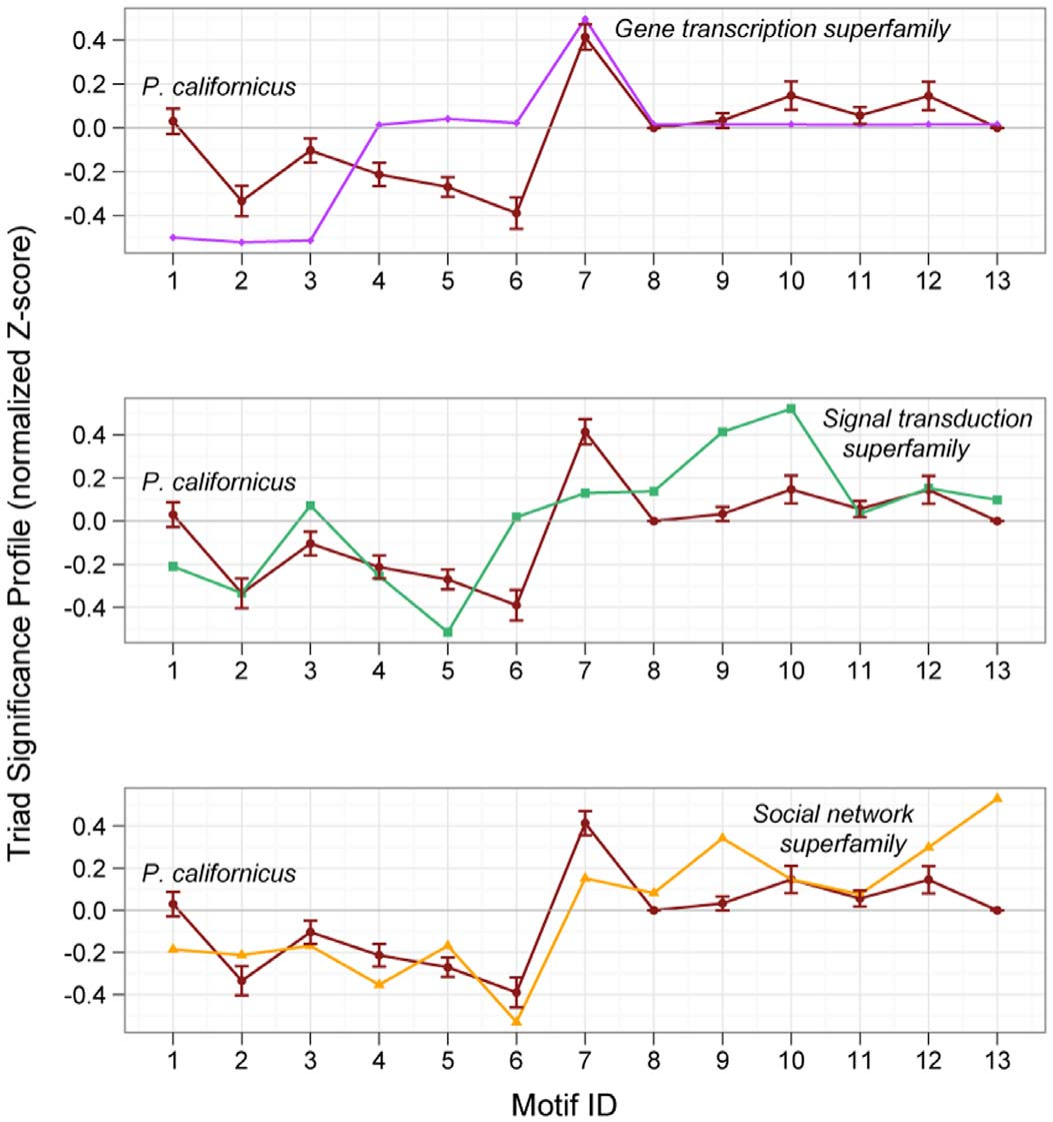
Social regulatory networks. Triad significance profiles compare the characteristic network motifs across a diverse range of network types and sizes by plotting standardized Z-scores which quantify the extent to which each subgraph is observed more or less frequently than expected in networks of a similar size and global structure but with randomized edge connections. The observed *P. californicus* social insect networks exhibit a distinct pattern of social regulatory structure combining elements found in previously identified major network superfamilies [22].

To evaluate the similarity of motif patterns across different networks and over time, we calculated the standardized Z-score for each subgraph [21] and constructed a triad significance profile (TSP) for each network (Figure 3). The TSP was consistent across all colony networks (Pearson’s r = 0.58±0.03, N = 66 comparisons, median p = 0.03). The motif distributions were also not significantly affected by the amount of time (26–130 s) used to populate interaction networks (Text S3).

When compared to the major network superfamilies [22], the combined motif signatures of our observed networks were somewhat more correlated with social networks (r = 0.68, p = 0.009) than the gene transcription (r = 0.48, p = 0.09) or the signal transduction (r = 0.60, p = 0.03) regulatory network superfamilies (Figure 3). Nevertheless, the correlation between colony and social networks was not significantly stronger than the correlation between colony and transcription networks (Δr = 0.20, n = 13, p = 0.49). The fully connected triad (motif 13: the social-clique motif), which is a defining characteristic of the human social network superfamily [22], was conspicuously uncommon in the *P. californicus* networks.

## Discussion

We compared the network motif profiles within social insect colony networks to the motif signatures for a range of technological and biological networks, including social networks. While the *P. californicus* networks exhibited scale-free structure and similarity with the general triad significance profile for the social network superfamily, the predominant motif within our colonies was the feed-forward loop. This interaction pattern is not typically identified with human social networks, but is involved in modulating information transmission in a range of regulatory networks across biological levels, including transcriptional regulation in *E. coli*, signal transduction between mammalian cells, and *C. elegans* synaptic wiring [29]–[31]. In contrast, the social-clique motif, characteristic of social attachment networks [21], [22], was absent in our *P. californicus* colony networks. The motif representation in *P. californicus* network structure supports the hypothesis that social network structure within these cohesive social groups has been selected to maximize colony-level function and/or efficiency rather than individual success. In other words, they are social regulatory networks, with key subgraph structures in common with regulatory networks across biological scales.

We suggest the motif signatures within social insect colonies may reflect selection for efficiency of directional information flow. Although all 13 subgraphs connect the same number of individuals, they vary in the costs of establishing and maintaining those connections. One way to compare efficiencies of interaction patterns is to evaluate the extent to which a particular subgraph maximizes the number of connected nodes (N) while minimizing costs of connectivity, in particular the number of edges as determined by interactions (I) and the resulting diameter (D) of the graph. In this way, subgraph efficiency (E) can be defined as E = N/(I*D). Applying this definition to the thirteen directed three-node subgraphs, calculated efficiencies range from 0.38 in motif 6, the motif of two mutual interactions, to 1.0 in motifs 7 and 8, the feed-forward loop and the three-cycle (Figure 2). The observation that the feed-forward loop is the characteristic motif signature among our colony networks suggests that efficiency of information transfer may be relevant to the patterns of connection among workers.

While this study has identified a number of intriguing features of communication patterns within social insect colonies, it also raises many new questions. One question to consider is how nest architectures may affect interaction dynamics. While the interaction patterns of individual ants may correlate with their spatial location within a nest [32], it is not clear whether location passively determines which type of interaction pattern individuals may be subjected to or engage in. Since ants tend to homeostatically regulate their densities [17] and exhibit spatial fidelity [33], we do not expect spatial position to be a causal factor influencing interaction patterns. However, given the substantial variation in labor-related specialization among workers within a colony, one factor that may be important is the extent to which individuals exhibit behavioral specialization for specific information-processing roles. An example of this kind of information-processing specialization has been identified in colonies of leaf-cutting ants, in which workers at the start of foraging may return to the nest unladen to increase the rate of information transmission to other workers within the nest [34]. By directly manipulating colony composition, we can empirically test hypotheses about the effects of spatial segregation and worker specialization. Additionally, by using different random models or generative network algorithms [5], the motif analysis method can be extended to test theoretical hypotheses about the temporal development and evolution of complex systems.

Animal groups exhibit an extreme range of social integration, from primarily solitary species that lack social cohesion to the complex interactions that shape superorganism species. To date there has been no network-based approach to separate out the very different mechanisms for network evolution across the diversity of social groups. Network motif analyses provide a new way to differentiate the interaction regimes under selection in social evolution. The markedly different subgraph characteristics of social insect and human societies open the field of network analysis for further exploration into the forces shaping social structure, function and evolution.

## Supporting Information

Movie S1 **Video recording of a paint-marked** ***Pogonomyrmex californicus*** **colony (QuickTime; 7.3 MB).**
(MOV)

Text S1 **Classification and identification of network motifs (PDF; 266 KB).**
(PDF)

Text S2 **Network degree distributions (PDF; 938 KB).**
(PDF)

Text S3 **Effect of analysis time on motif analysis (PDF; 578 KB).**
(PDF)
