# Supplementary material for: Information Processing in Social Insect Networks
Source: PLoS One. 2012 Jul 16;7(7):e40337. doi: 10.1371/journal.pone.0040337 (PMC3398002; doi:10.1371/journal.pone.0040337)
Supplement: Text S1 — Classification and identification of network motifs (PDF; 266 KB). (PDF) [file pone.0040337.s002.pdf]

**Supporting information, "Information processing in social insect colonies"**
**James S. Waters and Jennifer H. Fewell**

## Text S1: Classification and identification of network motifs

Network motif analysis is based on calculating the representation of specific subgraphs in observed networks and comparing these patterns to null models. In this paper, we focus on three-node subgraph representation patterns. Since these subgraphs are referred to using different terminology depending on the field, below we present the motif identification numbers as used in the regulatory motif literature [1,2] along with their corresponding identification codes from FANMOD [3], the isomorphism class numbers, and triad census names as used in R [4] and the igraph package [5]:

| Motif ID | Fanmod label | Isomorphism class number in R | Triad census name in R | Graph |
|---|---|---|---|---|
| 1 | 6 | 6 | 021D | A<-B->C |
| 2 | 36 | 2 | 021U | A->B<-C |
| 3 | 12 | 4 | 021C | A->B->C |
| 4 | 164 | 5 | 111D | A<->B<-C |
| 5 | 14 | 9 | 111U | A<->B->C |
| 6 | 78 | 10 | 201 | A<->B<->C |
| 7 | 38 | 7 | 030T | A->B<-C, A->C |
| 8 | 140 | 11 | 030C | A<-B<-C, A->C |
| 9 | 166 | 8 | 120D | A<-B->C, A<->C |
| 10 | 46 | 13 | 120U | A->B<-C, A<->C |
| 11 | 102 | 12 | 120C | A->B->C, A<->C |
| 12 | 174 | 14 | 210 | A->B<->C, A<->C |
| 13 | 238 | 15 | 300 | A<->B<->C, A<->C |

## Isomorphism classes, motifs, edge lists, and adjacency matrices

There are 16 isomorphism classes of three-node directed graphs. Note that there are only 13 of these graphs that connect all three nodes. Below, each of these is drawn and identified with the corresponding network motif ID.

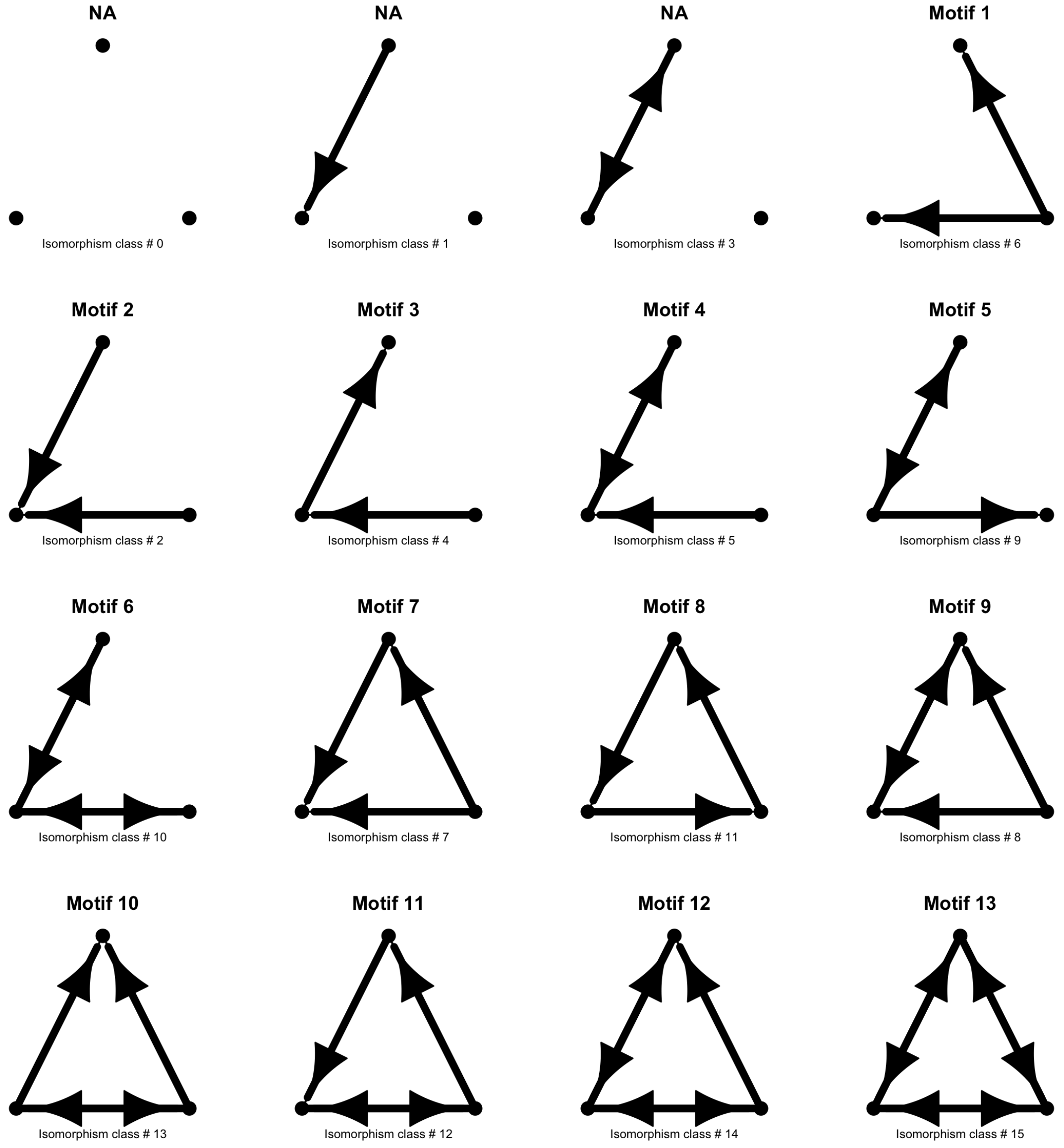

**Code to identify the 13 three-node network motifs and their corresponding isomorphism class in R:**

```
library(igraph)
par(mfrow=c(4,4))
par(mar=c(3,2,2,2))

iso.class <- c(0,1,3,6,2,4,5,9,10,7,11,8,13,12,14,15)

#      The isomorphism class numbers have been ordered according
#      to the sequence of network motif IDs following Milo et al.

labels <- c("NA", "NA", "NA", "Motif 1", "Motif 2", "Motif 3", "Motif 4", "Motif 5",
 "Motif 6", "Motif 7", "Motif 8", "Motif 9", "Motif 10", "Motif 11", "Motif 12", "Motif
 13")

for (i in 1:16){

      g <- graph.isocreate(size=3, number= iso.class[i], directed=T)

      g$layout <- layout.circle(g)
      g$layout [1,] <- c(-1, -1)
      g$layout [2,] <- c(0, 0.86)
      g$layout [3,] <- c(1, -1)

      V(g)$label <- NA
      E(g)$width=5
      E(g)$arrow.size=2
      E(g)$arrow.width=1
      E(g)$color="black"

     plot(g, vertex.color="black", main=labels[i])
     mtext(paste("Isomorphism class # ", iso.class[i], sep=""), side=1, line=0, cex=0.5)
     print(paste("Network Motif #", labels[i], sep=""))
     print(paste("Isomorphism class ", iso.class[i], sep=""))

     print("Edgelist")
     print(get.edgelist(g))
     print("Adjacency matrix")
     print(get.adjacency(g))

      }
```

**References**

1. Milo R, Itzkovitz S, Kashtan N, Levitt R, Shen-Orr S, et al. (2004) Superfamilies of evolved and designed networks. Science 303: 1538.
2. Milo R, Shen-Orr S, Itzkovitz S, Kashtan N, Chklovskii D, et al. (2002) Network Motifs: Simple Building Blocks of Complex Networks. Science 298: 824-827.
3. Wernicke S, Rasche F (2006) FANMOD: a tool for fast network motif detection. Bioinformatics 22: 1152.
4. R Development Core Team (2011) R: A language and environment for statistical computing. . R Foundation for Statistical Computing, Vienna, Austria. URL http://www.R-project.org/.
5. Csardi G, Nepusz T (2006) The igraph software package for complex network research. InterJournal, Complex Systems 1695: http://igraph.sf.net.
