# Supplementary material for: Information Processing in Social Insect Networks
Source: PLoS One. 2012 Jul 16;7(7):e40337. doi: 10.1371/journal.pone.0040337 (PMC3398002; doi:10.1371/journal.pone.0040337)
Supplement: Text S2 — Network degree distributions (PDF; 938 KB). (PDF) [file pone.0040337.s003.pdf]

Supporting information, “Information processing in social insect colonies”
James S. Waters and Jennifer H. Fewell

Text S2: Network Degree Distributions

For each colony network, we present the histograms for in- and out-degree as well as the scatterplots associated with estimating the exponent by OLS-regression for the power-law distribution describing the in- and out-degree scaling in each colony.

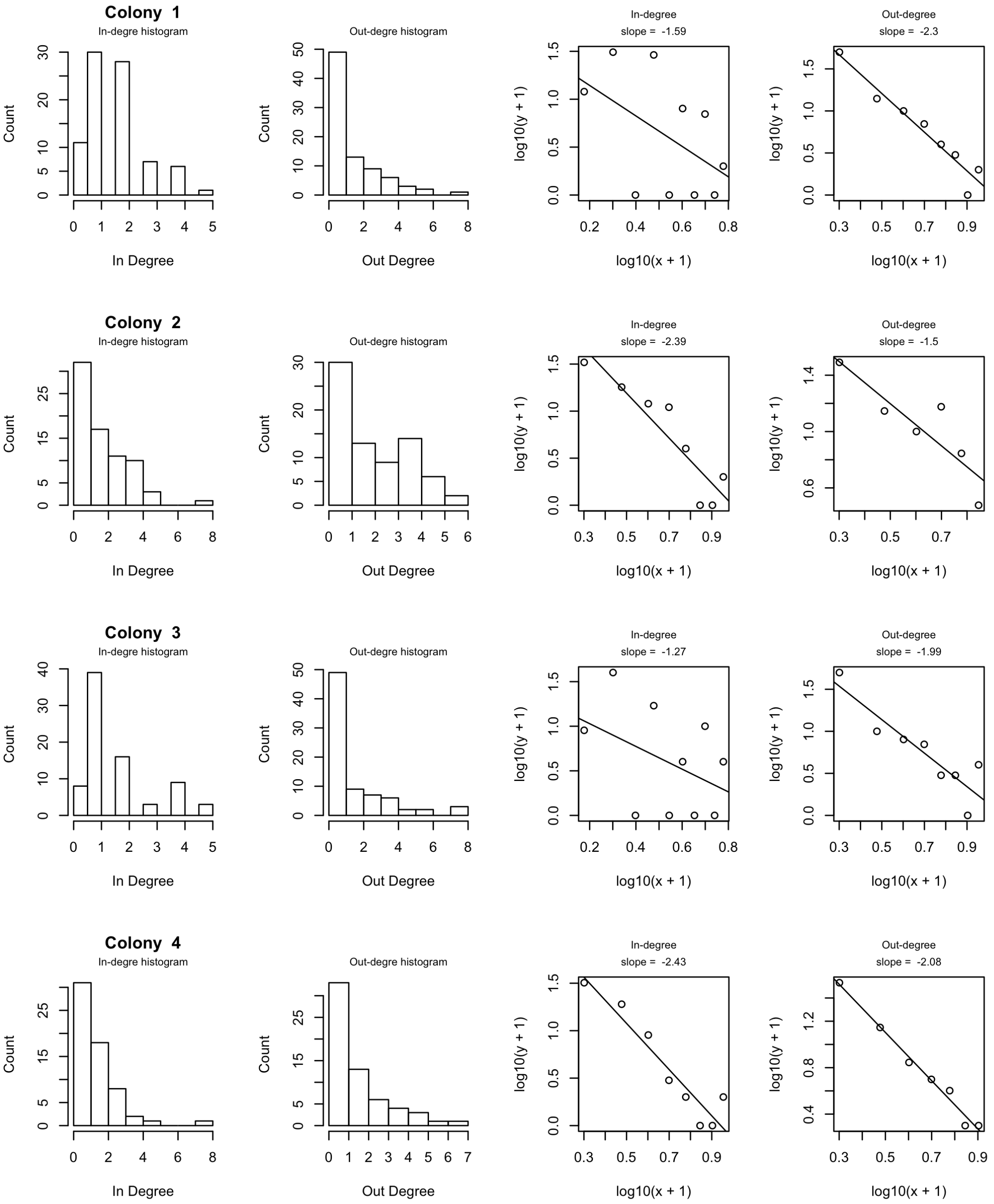
Colony 1
In-degre histogram
Count
In Degree
Out-degre histogram
Count
Out Degree
In-degree
slope = -1.59
log10(y + 1)
log10(x + 1)
Out-degree
slope = -2.3
log10(y + 1)
log10(x + 1)
Colony 2
In-degre histogram
Count
In Degree
Out-degre histogram
Count
Out Degree
In-degree
slope = -2.39
log10(y + 1)
log10(x + 1)
Out-degree
slope = -1.5
log10(y + 1)
log10(x + 1)
Colony 3
In-degre histogram
Count
In Degree
Out-degre histogram
Count
Out Degree
In-degree
slope = -1.27
log10(y + 1)
log10(x + 1)
Out-degree
slope = -1.99
log10(y + 1)
log10(x + 1)
Colony 4
In-degre histogram
Count
In Degree
Out-degre histogram
Count
Out Degree
In-degree
slope = -2.43
log10(y + 1)
log10(x + 1)
Out-degree
slope = -2.08
log10(y + 1)
log10(x + 1)

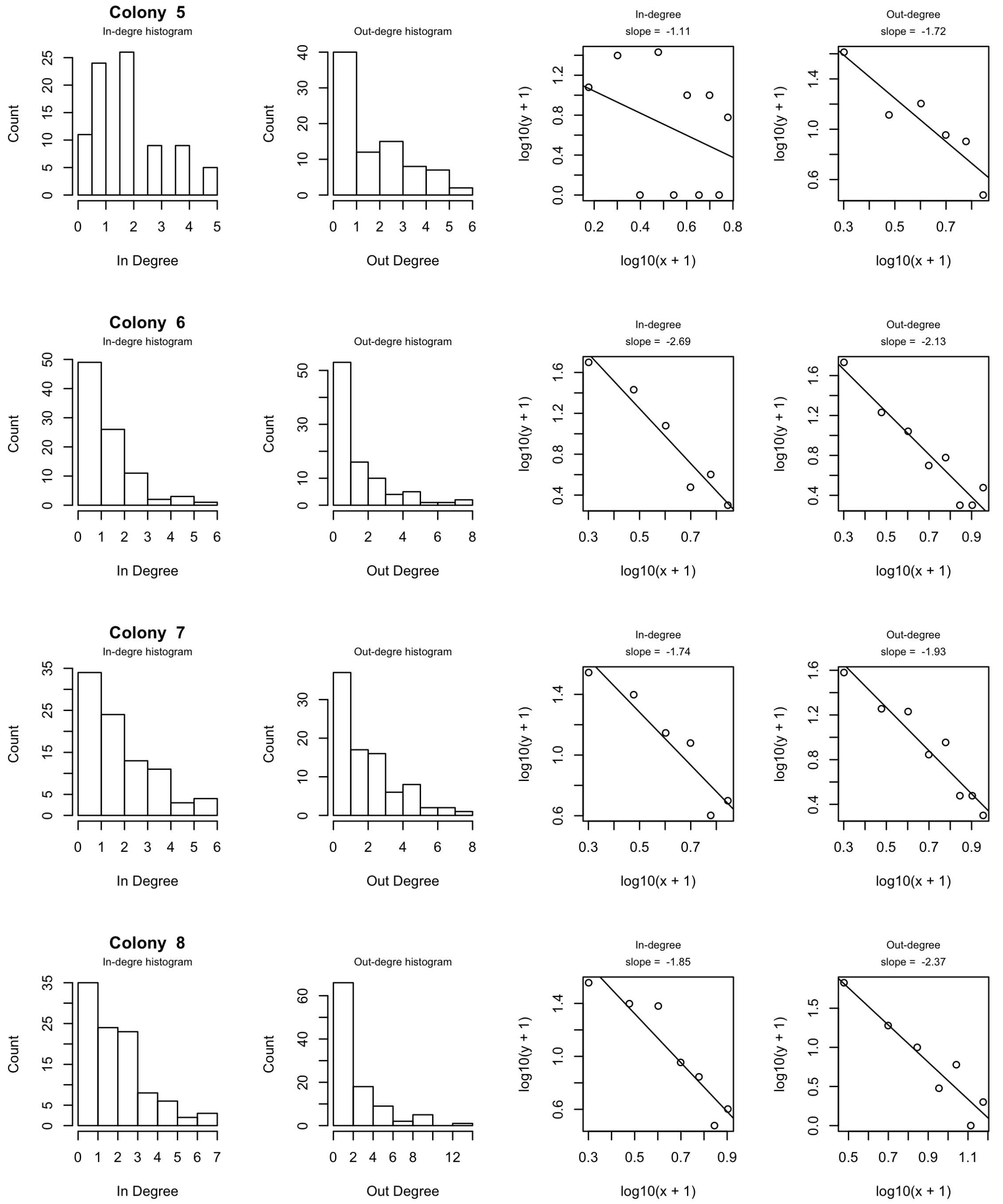

Colony 5
In-degre histogram
Count
In Degree
Out-degre histogram
Count
Out Degree
In-degree
slope = -1.11
log10(y + 1)
log10(x + 1)
Out-degree
slope = -1.72
log10(y + 1)
log10(x + 1)
Colony 6
In-degre histogram
Count
In Degree
Out-degre histogram
Count
Out Degree
In-degree
slope = -2.69
log10(y + 1)
log10(x + 1)
Out-degree
slope = -2.13
log10(y + 1)
log10(x + 1)
Colony 7
In-degre histogram
Count
In Degree
Out-degre histogram
Count
Out Degree
In-degree
slope = -1.74
log10(y + 1)
log10(x + 1)
Out-degree
slope = -1.93
log10(y + 1)
log10(x + 1)
Colony 8
In-degre histogram
Count
In Degree
Out-degre histogram
Count
Out Degree
In-degree
slope = -1.85
log10(y + 1)
log10(x + 1)
Out-degree
slope = -2.37
log10(y + 1)
log10(x + 1)

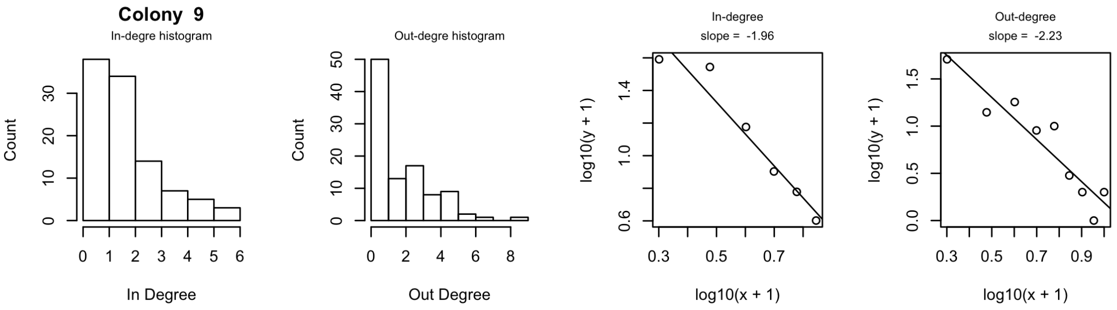
Colony 9
In-degre histogram
Count
In Degree
Out-degre histogram
Count
Out Degree
In-degree
slope = -1.96
log10(y + 1)
log10(x + 1)
Out-degree
slope = -2.23
log10(y + 1)
log10(x + 1)

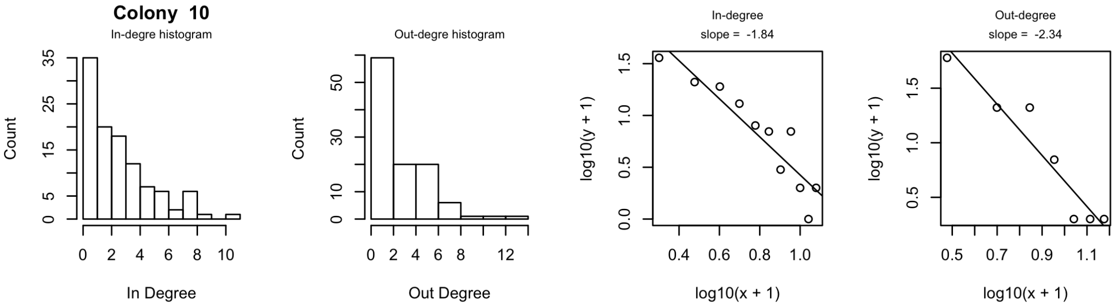
Colony 10
In-degre histogram
Count
In Degree
Out-degre histogram
Count
Out Degree
In-degree
slope = -1.84
log10(y + 1)
log10(x + 1)
Out-degree
slope = -2.34
log10(y + 1)
log10(x + 1)

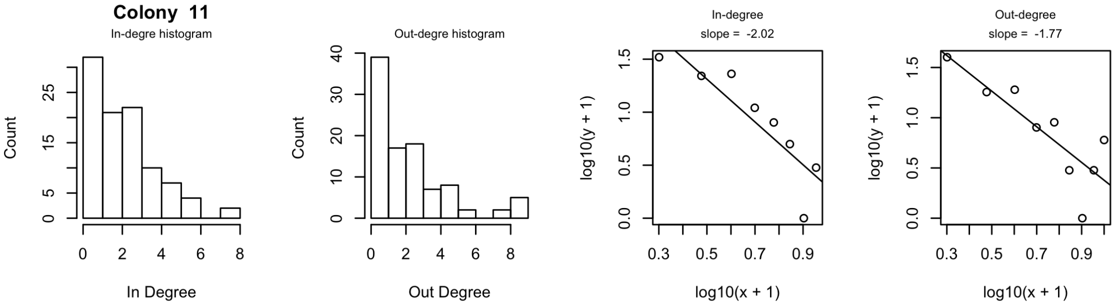
Colony 11
In-degre histogram
Count
In Degree
Out-degre histogram
Count
Out Degree
In-degree
slope = -2.02
log10(y + 1)
log10(x + 1)
Out-degree
slope = -1.77
log10(y + 1)
log10(x + 1)

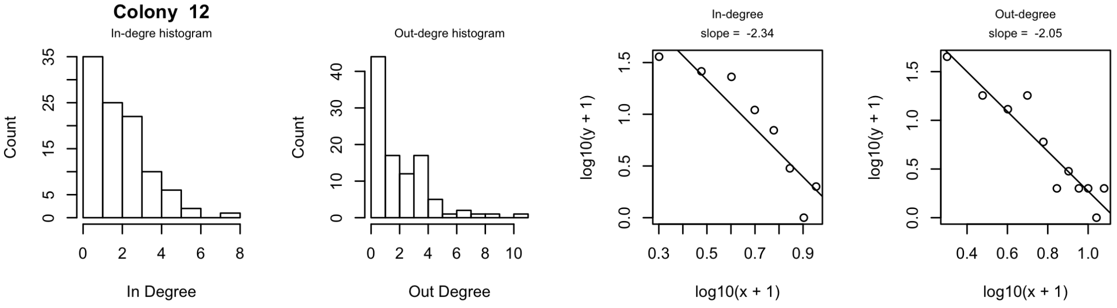
Colony 12
In-degre histogram
Count
In Degree
Out-degre histogram
Count
Out Degree
In-degree
slope = -2.34
log10(y + 1)
log10(x + 1)
Out-degree
slope = -2.05
log10(y + 1)
log10(x + 1)
