# Supplementary material for: Information Processing in Social Insect Networks
Source: PLoS One. 2012 Jul 16;7(7):e40337. doi: 10.1371/journal.pone.0040337 (PMC3398002; doi:10.1371/journal.pone.0040337)
Supplement: Text S3 — Effect of analysis time on motif analysis (PDF; 578 KB). (PDF) [file pone.0040337.s004.pdf]

Supporting information, "Information processing in social insect colonies"
James S. Waters and Jennifer H. Fewell

Text S3: Effect of analysis time on subgraph Z-scores

The effect of analysis time on motif representation was examined by constructing cumulative networks spanning 30-130 seconds of whole-colony interaction. These networks exhibited a considerable range in size from 83 individuals and 136 interactions in a network based on 26 seconds of recorded behavior to 129 individuals engaging in 1117 interactions in a network based on 130 seconds of recorded behavior.

The Z-score is defined as the ratio of the difference in subgraph density between an observed network and its average density in a set of 10,000 randomized networks divided by the standard deviation of the subgraph's density in the randomized networks. Although there are visible trends in which the Z-scores associated with individual subgraphs (IDs 1-13) either increase or decrease with the amount of time analyzed (and network size), none of the linear regressions were significant (p-values ranging from 0.20-0.99), suggesting that the method of motif analysis is robust with respect to the amount of time analyzed.

The figure below shows a scatterplot of the Z-scores for each subgraph as a function of the amount of time analyzed in constructing the interaction network. The table below gives the estimates and standard error for intercept and slope as well as t-score and p-value for the slope of each of the regression models fitting subgraph Z-score as a function of the amount of time used to construct the respective networks.

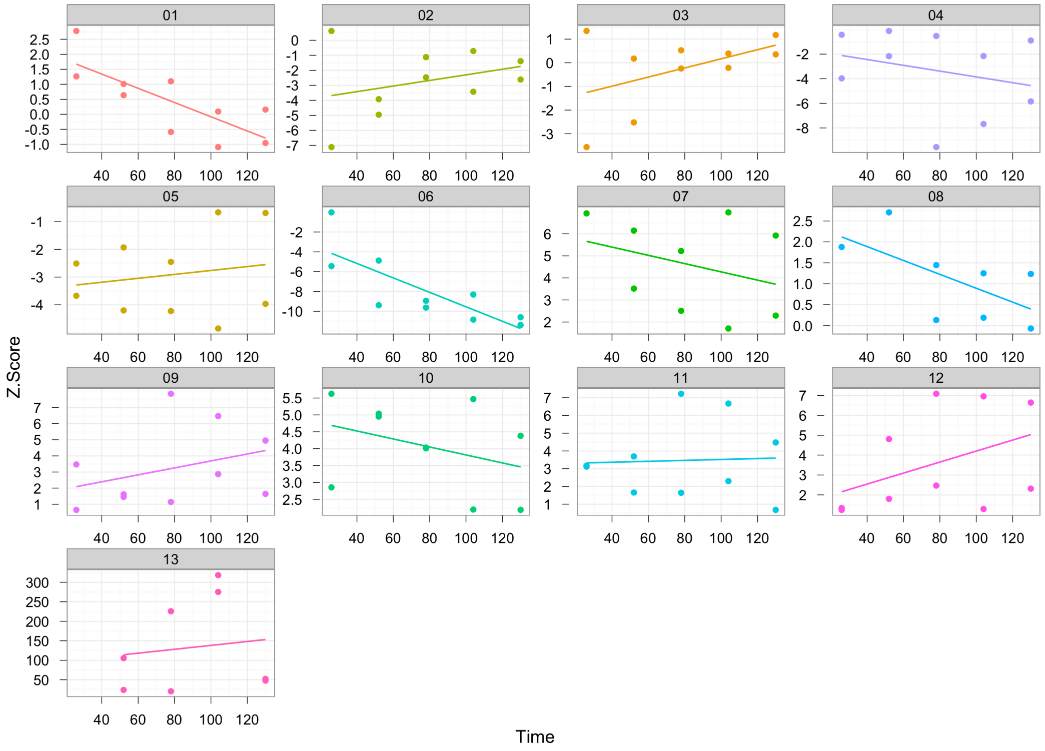

Regression summary statistics:

Subgraph	Intercept	Slope	t	Pr (>|t|)
1	2.289 ± 23.685	-0.024 ± 0.275	-0.086	0.9314
2	-6.459 ± 33.496	0.019 ± 0.275	0.068	0.9459
3	-4.050 ± 33.496	0.019 ± 0.275	0.07	0.9442
4	-3.791 ± 33.496	-0.024 ± 0.275	-0.086	0.932
5	-5.758 ± 33.496	0.007 ± 0.275	0.026	0.9795
6	-4.544 ± 33.496	-0.073 ± 0.275	-0.266	0.7911
7	3.856 ± 36.811	-0.019 ± 0.311	-0.06	0.9522
8	0.261 ± 39.061	-0.017 ± 0.330	-0.05	0.96
9	-0.759 ± 33.496	0.022 ± 0.275	0.078	0.9376
10	2.7078 ± 33.496	-0.012 ± 0.275	-0.043	0.9658
11	0.973 ± 33.496	0.003 ± 0.275	0.009	0.9925
12	-0.851 ± 33.496	0.028 ± 0.275	0.101	0.92
13	86.039 ± 44.022	0.499 ± 0.389	1.284	0.2022
